# Exploring the relationship between calcitonin, ionized calcium, and bone turnover in cats with and without naturally occurring hypercalcemia

**DOI:** 10.3389/fvets.2024.1399942

**Published:** 2024-06-03

**Authors:** Evangelia Maniaki, Carmen Pineda, Angie Hibbert, Natalie Finch

**Affiliations:** ^1^Bristol Veterinary School, University of Bristol, Langford, Bristol, United Kingdom; ^2^Department of Animal Medicine and Surgery, University of Cordoba, Córdoba, Spain; ^3^The Feline Centre, Langford Vets, University of Bristol, Langford, Bristol, United Kingdom; ^4^Bristol Renal, University of Bristol, Bristol, United Kingdom

**Keywords:** cat, feline, calcitonin, calcium, hypercalcemia, idiopathic

## Abstract

**Objectives:**

This case-control study aimed to evaluate calcitonin response in naturally occurring hypercalcemia in cats and assess the relationships between calcitonin and ionized calcium (iCa) and examine relationships between calcitonin, iCa and bone turnover.

**Methods:**

Hypercalcemic cats (persistently increased iCa concentration [>1.40 mmol/l]) were identified retrospectively via a medical database search; additional hypercalcemic and normocalcemic cats were recruited prospectively. Data regarding routine biochemical and urine testing, diagnostic imaging and additional blood testing were obtained. Serum alkaline phosphatase (ALP) activity was used as a marker of bone turnover. Serum calcitonin concentration was analyzed using a previously validated immunoradiometric assay. Hypercalcemic cats with an increased calcitonin concentration (>0.9 ng/L) were termed responders. Group comparisons were performed using a Mann-Whitney test for continuous variables and a χ2 test for categorical variables. Spearman’s correlation coefficient was used to examine the relationships between calcitonin, iCa and ALP.

**Results:**

Twenty-six hypercalcemic and 25 normocalcemic cats were recruited. Only 5/26 (19.2%) of the hypercalcemic cats were identified as responders, and all were diagnosed with idiopathic hypercalcemia. There was no significant correlation between the concentrations of calcitonin and iCa (*p* = 0.929), calcitonin and ALP (*p* = 0.917) or iCa and ALP (*p* = 0.678) in hypercalcemic cats, however, a significant negative correlation was observed between calcitonin and ALP (*p* = 0.037) when normocalcemic and hypercalcemic cats with an elevated calcitonin concentration were analyzed together.

**Discussion:**

The expected increase in calcitonin concentration was present in only a small subset of hypercalcemic cats; no correlation was found between iCa and calcitonin concentration. The inverse relationship between calcitonin and ALP in cats with increased calcitonin concentrations suggests that the ability of calcitonin to correct hypercalcemia may be related to the degree of bone turnover.

## 1 Introduction

Calcium homeostasis is tightly regulated by interactions between parathyroid hormone (PTH), calcitriol (the active form of vitamin D) and calcitonin. Calcitonin is synthesized by the parafollicular or C cells in the thyroid gland and secreted in response to hypercalcemia. Calcitonin restores normocalcemia by predominantly inhibiting osteoclastic bone resorption (1–3), but also by decreasing renal tubular reabsorption of calcium (4). The ability of calcitonin to decrease the magnitude of hypercalcemia has been established both in azotemic and non-azotemic animal models (5–8), but it is not yet clear whether its significance is greater in one state compared to the other. Further, it has been suggested in some species that the ability of calcitonin to reduce circulating calcium concentration is related to the degree of bone turnover (9, 10). The role of calcitonin in calcium regulation has not been fully elucidated in most mammals and studies investigating the precise function of calcitonin in cats are lacking, however, its function is acknowledged to be conserved across species (11).

Idiopathic hypercalcemia is one of the most commonly reported causes of naturally occurring hypercalcemia, reported in 48–52% of hypercalcemic cats (12). Chronic kidney disease (CKD) is also common (13), reported in 25.4–35% (14, 15) of hypercalcemic cats. However, the relationship between CKD and hypercalcemia is complex as hypercalcemia can both cause renal damage and develop as a consequence of CKD (16). Hypercalcemia of malignancy accounts for 29.6–35% of hypercalcemia in cats (14, 15, 17). Other causes of hypercalcemia in cats include hypervitaminosis D, primary hyperparathyroidism, granulomatous disease, hypoadrenocorticism, and hyperthyroidism (14, 15, 17–24).

A previous study in cats with experimentally induced hypercalcemia identified a group of cats that failed to increase calcitonin production in response to hypercalcemia (25). Although C cells were also present in these cats, their numbers were significantly lower (total number of calcitonin-positive C cells and mean number of C cells per field), and their functional ability to produce calcitonin is unknown. These cats were termed “non-responders,” and this finding suggests that a subgroup of cats may not increase calcitonin concentration in response to ionized hypercalcemia. Similarly, in a recent study exploring calcitonin response to naturally occurring ionized hypercalcemia in cats with azotemic CKD (26), only a third of the cats had a measurable increase in calcitonin concentration.

The aims of this study were (a) to compare serum calcitonin concentrations between normocalcemic and hypercalcemic cats, (b) to evaluate calcitonin response in cats with naturally occurring hypercalcemia, and (c) to investigate the relationship between calcitonin, ionized calcium (iCa) and bone turnover in cats. We hypothesized that there would be no difference in the serum calcitonin concentrations between normocalcemic and hypercalcemic cats.

## 2 Materials and methods

This case–control study was approved by the University of Bristol’s Animal Welfare and Ethical Review Body (VIN/15/050).

The medical database at Langford Vets’ Small Animal Hospital, University of Bristol, UK was searched to identify hypercalcemic cats referred to the Feline Centre between January 2011 and June 2015. Additional hypercalcemic and normocalcemic (control) cats were identified at the time of presentation to the Feline Centre between July 2015 and June 2016. Hypercalcemia was defined as persistently increased iCa concentration (>1.40 mmoL/L [reference interval (RI): 1.10–1.40 mmoL/L]) on two or more repeated samples. Normocalcemia was defined as an iCa concentration and/or a total calcium (tCa) concentration (RI: 2.30–2.50 mmoL/L) within the RI. iCa was measured in heparinized blood immediately following collection using an ion-selective electron analyzer (RAPIDPoint^®^ 500). The control cat population included normocalcemic cats without disease or receiving medication known to affect calcium or calcitonin homeostasis.

Serum concentrations of tCa, creatinine, urea, phosphate, alkaline phosphatase (ALP) and urine specific gravity (USG) were measured at a commercial reference laboratory (Langford Diagnostic Laboratories, Langford Vets, Bristol). Urine was either voided voluntarily and collected using non–absorbent cat litter or collected via cystocentesis. Additional testing in individual hypercalcemic cases was undertaken according to the clinician’s decision (an ECVIM [European College of Veterinary Internal Medicine] diplomate or diplomat in training), and this was also recorded in the study. This included serum measurement of PTH, parathyroid hormone-related protein (PTHrP), calcitriol, basal cortisol, and findings from imaging modalities (radiography, ultrasound, computed tomography, magnetic resonance imaging). Clinical notes were reviewed by an ECVIM diplomate to obtain the clinician’s final diagnosis for both hypercalcemic (cases) and normocalcemic (controls) cats.

Serum biochemistry and measurement of PTH, PTHrP, and calcitriol was submitted to a commercial reference laboratory (Langford Vets Diagnostic Laboratories). Cats with a concentration below the lower limit of detection of the PTH assay (1 pmol/L) were assigned a value of 1 pmol/L. Serum ALP activity was used as a marker of bone turnover (27, 28). Residual serum samples that had been stored at –80°C for an average of 416 days (range: 90 to 742 days) were transported frozen to the University of Cordoba, Spain for measurement of calcitonin concentration.

Measurement of calcitonin concentration was undertaken using a human immunoradiometric assay (Scantibodies Laboratory Inc., Santee, California) previously validated for cats (25). Cats with calcitonin concentration below the lower limit of detection of the assay (0.9 ng/L or 0.9 pg/mL) were assigned a value of 0.9 ng/L. Hypercalcemic cats were further grouped into responders and non-responders based on whether they had a calcitonin concentration above the lower limit of detection.

Statistical analysis was conducted using SPSS (version 27.0, IBM Corporation, United States) and GraphPad Prism (version 8.1.2, GraphPad Software, United States). Data were assessed for normality using the Kolmogorov–Smirnov test and by visual inspection of graphical plots. Non-parametric statistical tests were applied to the data. Group comparisons were performed using a Mann–Whitney test for continuous variables and a χ2 test or Fisher’s exact test for categorical variables. The correlation between iCa and calcitonin, iCa and ALP activity, and calcitonin and ALP activity were examined using Spearman’s correlation coefficient (ρ). Statistical significance was set at *p* < 0.05, and an exact (2-tailed) significance is reported.

## 3 Results

**Figure 1:**
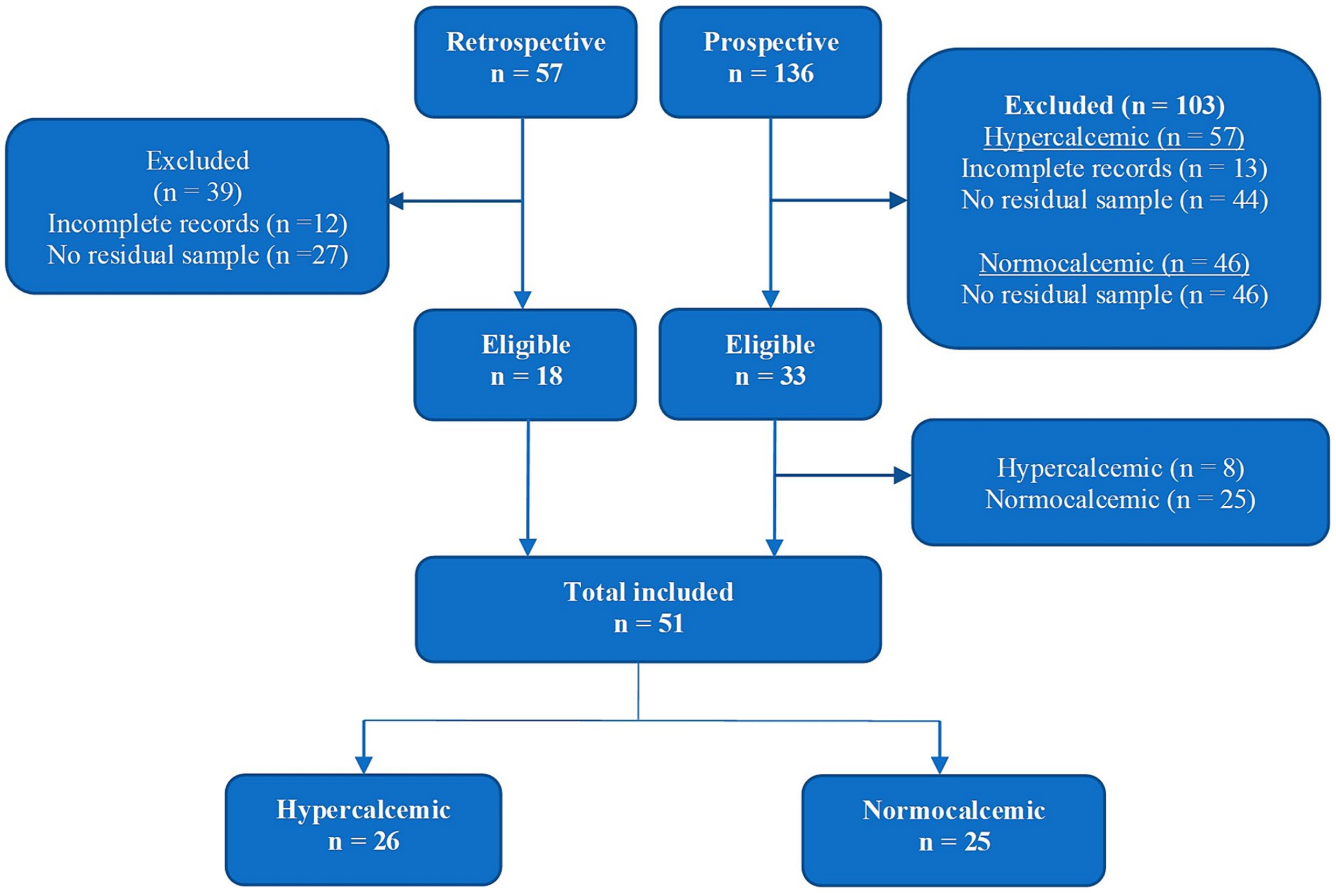
Recruitment and enrolment flowchart.

Cats were recruited both retrospectively (medical database search between January 2011 and June 2015) and prospectively (recruitment between July 2015 and June 2016) as illustrated in Figure 1. Normocalcemic cats were only recruited prospectively. The total study population comprised of 51 cats: 26 hypercalcemic and 25 normocalcemic.

The median age for all cats was 7.77 years with a range of 0.8 to 17 years. There were 26 male cats, of which one was entire, and 25 female cats, of which two were entire. The cats were of the following breeds: domestic shorthair (*n* = 37), British shorthair (*n* = 1), two each of domestic longhair, Burmese, Maine Coon and Persian, and one each of Bengal, Birman, Selkirk Rex, Siberian, and Snowshoe.

Twenty-five normocalcemic cats were included in the control group. Of these cats, five (20%) had feline lower urinary tract disease (FLUTD), four (16%) endocrinological disease (three with hyperthyroidism, one with diabetes mellitus), three (12%) cardiac disease (hypertrophic cardiomyopathy), two (8%) gastrointestinal disease (gastroenteritis and pancreatitis), two (8%) neoplastic disease (suspected lymphoma), two (8%) neurological disease (idiopathic vestibular syndrome), two (8%) trauma (one with pelvic fracture and one with pneumothorax), and one (4%) infectious disease (virulent systemic Feline Calicivirus). Four (16%) cats had inconclusive diagnoses.

A comprehensive review of medical history, including dietary information, was conducted for all hypercalcemic cats. All cats were fed commercially available age-appropriate diets (e.g., adult, senior), with no additional vitamin D supplementation or history of accidental vitamin D ingestion. Furthermore, no cats were fed a renal diet. Of the 26 cats with naturally occurring hypercalcemia, 13 (50.0%) were diagnosed with idiopathic hypercalcemia, followed by seven (26.9%) cats with CKD. According to the International Renal Interest Society (IRIS) staging system (29), one cat had stage 1 CKD, four cats had stage 2 CKD, and two cats had stage 3 CKD. Before their diagnosis, all cats were fed age-appropriate commercial diets. Finally, two cats (7.7%) were diagnosed with neoplasia (lymphoma and thyroid carcinoma), and one cat with granulomatous disease (3.8%). A conclusive etiology was not established in three cats (11.5%); the first was either a granuloma or idiopathic hypercalcemia, the second granuloma or neoplasia, and the third primary hyperparathyroidism or idiopathic hypercalcemia.

There was no significant difference in signalment variables between controls and hypercalcemic cats (Table 1).

**Table 1:** Group comparisons for signalment variables in hypercalcemic (*n* = 26) and control (*n* = 25) cats.

Variables		All cats, *n* (%)	Hypercalcemic cats, *n* (%)	Control cats, *n* (%)	*p* value
Combined sex/neuter status	Male neutered	25 (49.0%)	13 (50.0%)	12 (48.0%)	*p* = 0.889
	Male entire	1 (2.0%)	0 (0%)	1 (4.0%)	
	Female neutered	23 (45.1%)	13 (50.0%)	10 (40.0%)	
	Female entire	2 (3.9%)	0 (0%)	2 (8.0%)	
Breed category	DSH and DLH	39 (76.5%)	18 (69.2%)	21 (84.0%)	*p* = 0.301
	Purebred	12 (23.5%)	8 (30.8%)	4 (16.0%)	
Age in years (range)		7.7 (0.8–17.0)	7.1 (1–17.0)	8.1 (0.8–16.2)	*p* = 0.611

Categorical variables are presented as number (*n*) and % of cats and continuous variables as median (range). Group comparisons were performed using a χ2 test or Fisher’s exact test for categorical variables and a Mann–Whitney test for continuous variables. DSH, domestic shorthair; DLH, domestic longhair.

**Figure 2:**
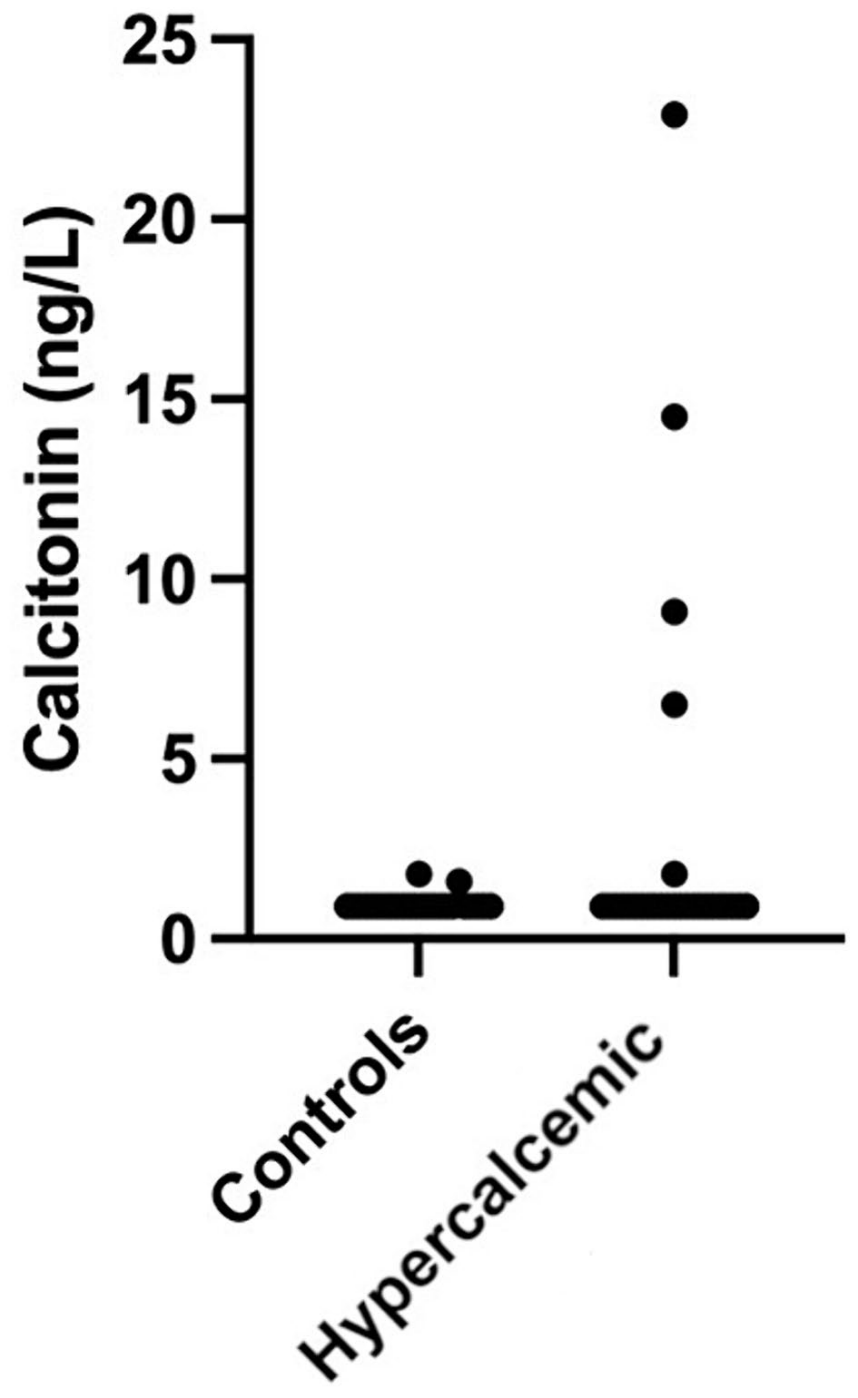
Calcitonin concentration in hypercalcemic (*n* = 26) and control (*n* = 25) cats. Concentrations were 1.6 and 1.7 ng/mL for the two control cats and 1.8, 6.5, 9.1, 14.5, and 22.9 ng/mL for the five hypercalcemic cats.

One control cat had a tCa concentration above the reference range (3.4 mmol/L); however, both iCa and calcitonin concentrations were within normal limits. Increased calcitonin concentration was observed in five (19.2%) hypercalcemic and two (8%) control cats (Figure 2). Calcitonin concentration did not differ between hypercalcemic and control cats. However, in addition to iCa and tCa, a significant statistical difference was identified in all other clinicopathological variables, including ALP, creatinine, urea, phosphate, and USG (Table 2).

**Table 2:** Clinicopathological variables of hypercalcemic (*n* = 26) and control (*n* = 25) cats.

	Hypercalcemic cats		Control cats		
Variable (RI)	Median (range)	*n*	Median (range)	*n*	*p* value
Ionized Calcium (1.10–1.40 mmol/L)	1.60 (1.43–2.29)	26	1.22 (1.04–1.37)	17	*p* = <0.0001*
Total Calcium (2.30–2.50 mmol/L)	3.28 (2.6–4.0)	26	2.30 (2.0–3.4)	12	*p* = <0.0001*
Calcitonin (<0.9 ng/L)	0.9 (0.9–22.9)	26	0.9 (0.9–1.8)	25	*p* = 0.269
ALP (15–60 IU/L)	23.50 (7–90)	26	26.50 (20–227)	6	*p* = <0.0001*
Creatinine (133–175 μmol/L)	143 (53–352)	25	121 (54–447)	13	*p* = <0.0001*
Urea (6.5–10.5 mmol/L)	8.9 (4.8–34.3)	25	8.1 (5.1–41.8)	14	*p* = <0.0001*
Phosphate (0.95–1.55 mmol/L)	1.38 (0.9–2.54)	25	1.46 (1.1–3.09)	7	*p* = <0.0001*
USG (>1.035)	1.030 (1.012–1.055)	16	1.022 (1.014–1.050)	7	*p* = <0.0001*

RI, reference interval; USG, urine specific gravity. Statistically significant results are designated with *.

Additional blood testing that was undertaken in the diagnostic work-up of the hypercalcemic cats was unremarkable and is presented in Table 3.

**Table 3:** Additional investigations in hypercalcemic cats (*n* = 26).

Variable (RI)	*n*	Median (range)	*n* (%) of cats outside RI
PTH (<4.2 pmol/L)	13	1.0 (1.0–3.0)	0 (0.0%)
PTHrP (<0.5 pmol/L)	12	0.1 (0.1–0.3)	0 (0.0%)
Calcitriol (22–137 pmol/L)^†^	6	12.5 (12–137)	0 (0.0%)
Cortisol (28–200 nmol/L)	4	83.5 (60–113)	0 (0.0%)

PTH, parathyroid hormone; PTHrP, parathyroid hormone-related protein; RI, reference interval. ^†^Reference interval derived from Barber et al. (30).

Only 5/28 (17.9%) cats had an increased calcitonin concentration in response to hypercalcemia, and all were diagnosed with idiopathic hypercalcemia. No significant difference in signalment was found between hypercalcemic cats that increased their calcitonin concentration (responders) and cats that did not (non-responders) (Table 4).

**Table 4:** Group comparisons for signalment data in hypercalcemic cats (*n* = 26) based on response (*n* = 5) or lack of response (*n* = 21) to ionized hypercalcemia.

Variables		Hypercalcemic cats, *n* (%)	Responder cats, *n* (%)	Non–responder cats, *n* (%)	*p* value
Combined sex/neuter status	Male Neutered	13 (50.0%)	2 (40.0%)	11 (52.4%)	*p* = 0.626
	Female Neutered	13 (50.0%)	3 (60.0%)	10 (47.6%)	
Breed category	DSH and DLH	18 (69.2%)	3 (60.0%)	15 (71.4%)	*p* = 0.315
	Purebred	8 (30.8%)	2 (40.0%)	6 (28.6%)	
Age in years (range)		7.1 (1–17.0)	3.27 (2.6–17.0)	7.77 (1.0–16.5)	*p* = 0.820

Categorical variables are presented as number (*n*) and % of cats and continuous variables as median (IQR). Group comparisons were performed using a χ2 test or Fisher’s exact test for categorical variables and a Mann–Whitney test for continuous variables. DSH, domestic shorthair; DLH, domestic longhair.

There was no significant difference in any of the clinicopathological variables between hypercalcemic responder and non-responder cats (Table 5) except for USG which was significantly higher in responder compared to non-responder cats (*p* = 0.034).

**Table 5:** Clinicopathological variables of hypercalcemic cats (*n* = 26) based on response (*n* = 5) or lack of response (*n* = 21) to ionized hypercalcemia.

	Responder cats (*n* = 5)	Non–responder cats (*n* = 21)	
Variable (RI)	Median (range)	Median (range)	*p* value
Ionized Calcium (1.10–1.40 mmol/L)	1.59 (1.53–1.84)	1.60 (1.43–2.29)	*p* = 0.896
Total Calcium (2.30–2.50 mmol/L)	3.25 (3.1–3.8)	3.29 (2.6–4.0)	*p* = 1.000
ALP (15–60 IU/L)	27.50 (11–34)	23.50 (7–90)	*p* = 0.969
Creatinine (133–175 μmol/L)	143.0 (111.0–215.0)	141.0 (53.0–352.0)	*p* = 0.786
Urea (6.5–10.5 mmol/L)	9.1 (7.3–19.7)	8.85 (4.8–34.3)	*p* = 0.786
Phosphate (0.95–1.55 mmol/L)	1.38 (1.22–1.56)	1.39 (0.9–2.54)	*p* = 0.892
USG (>1.035)	1.050 (1.026–1.055)	1.025 (1.017–1.050)	*p* = 0.034*

ALP, alkaline phosphatase; RI, reference interval; USG, urine specific gravity. Statistically significant results are designated with *.

Interestingly, 2/25 (8%) control cats also had an increased calcitonin concentration; these were a 16-year-old cat diagnosed with severe restrictive cardiomyopathy and a 9-year-old cat with hyperthyroidism. When examining the correlations between serum calcitonin, blood iCa and serum ALP concentration, a negative significant correlation was detected between serum calcitonin and serum ALP concentration (ρ = −0.9, *p* = 0.037) in cats with increased calcitonin concentration (>0.9 ng/L) (Table 6). No other significant correlations were detected.

**Table 6:** Correlation analyses in all cats (*n* = 51), hypercalcemic cats (*n* = 26) and cats with increased calcitonin concentration (*n* = 7).

	All cats (*n* = 51)		Hypercalcemic cats (*n* = 26)		Cats with increased calcitonin concentration (>0.9 ng/L, *n* = 7)	
	ρ	*p* value	ρ	*p* value	ρ	*p* value
Calcitonin – iCa	0.018	0.929	0.018	0.929	−0.400	0.505
Calcitonin – ALP	−0.022	0.917	−0.022	0.917	−0.900	0.037*
iCa – ALP	−0.089	0.677	−0.089	0.678	0.800	0.200

Correlations were examined using Spearman’s correlation coefficient (ρ). ALP, alkaline phosphatase; iCa, ionized calcium. Statistically significant results are designated with *.

## 4 Discussion

Previous studies have only evaluated calcitonin response in cats with experimentally induced hypercalcemia and in cats with concurrent naturally occurring hypercalcemia and azotemic CKD (25, 26). This is the first study in which calcitonin response has been evaluated in cats with naturally occurring hypercalcemia of differing etiologies and was additionally compared to that of normocalcemic control cats.

Similarly low calcitonin concentrations for which cats would be considered non-responders have been reported in previous studies of experimentally induced hypercalcemia in healthy cats and hypercalcemic cats with azotemic CKD (25, 26). This finding is not unique to cats; failure to increase calcitonin secretion in response to hypercalcemia has been reported in normal and azotemic humans (31), as well as in studies with experimentally induced hypercalcemia in humans (32, 33) and rats (34). Interestingly, the percentage of non-responder cats in this study (80.8%) is higher than that reported in humans (between 15 and 33% in men and 44% in women) (31–33). In agreement with a previous study (26), there was no significant relationship between serum calcitonin and blood iCa concentration in hypercalcemic cats, with responders demonstrating as severe hypercalcemia as non-responder cats. Although the observed degree of ionized hypercalcemia was similar to that reported in cats with experimentally induced hypercalcemia and in cats with concurrent naturally occurring hypercalcemia and CKD (25, 26), only 19.2% of cats were classified as responders in this study compared to 46.2 and 33.3%, respectively.

All five responder cats in this study were diagnosed with idiopathic hypercalcemia. The mechanism behind the development of idiopathic hypercalcemia, one of the most commonly identified diagnoses in hypercalcemic cats, has not been elucidated (14, 35). One potential explanation may be related to genetic factors. The homeostatic action of the calcium-sensing receptor (CaSR) has been examined in CaSR knockout mice models (36, 37), demonstrating that it regulates PTH and calcitonin secretion from the parathyroid glands and C cells of the thyroid gland, respectively. In humans, mutations in the CaSR cause familial hypocalciuric hypercalcemia in heterozygotes and neonatal severe hyperparathyroidism in homozygotes, resulting in inappropriately normal or elevated PTH concentrations in the face of hypercalcemia (38). In a recent study investigating the CaSR in cats with CKD with and without concurrent hypercalcemia, an association was found between 1/12 CaSR single nucleotide polymorphisms (SNPs) and PTH concentration, but not iCa concentration (39); however, calcitonin concentration was not measured. It is therefore possible that some cats with hypercalcemia have unidentified CaSR SNPs that account for the lack of calcitonin response in the face of hypercalcemia. The presence of CaSR SNPs could also explain the disparity in the number of calcitonin-positive C cells between responders and non-responders to experimental hypercalcemia (25). Another possible explanation relates to the chronicity of hypercalcemia. Experimental studies in rats with chronic hypercalcemia demonstrated exhaustion of the calcitonin content of the thyroid gland and a diminished calcitonin response (34). This finding could account for the small number of responders observed in this study as well as in the study of hypercalcemic cats with CKD (26). It is also unclear why two normocalcemic cats had an increased calcitonin concentration. A possible explanation would be that their ionized calcium had transiently increased to stimulate calcitonin production but was maintained within normal limits by calciuresis (3, 8); unfortunately, measurement of the fractional excretion of calcium was not possible.

Creatinine, urea, phosphate, USG and ALKP were statistically different between hypercalcemic and control cats, most likely as a result of 26.9% of hypercalcemic cats being diagnosed with CKD. Only USG was significantly higher in responders compared to non-responder cats. Urine specific gravity is influenced by sex, diet type, fasting status and drinking avidity (40), as well as renal status (41). In human studies, C-cell hyperplasia and secondary hypercalcitoninemia occur only in human patients with advanced stage CKD (42). Most cats with CKD in the present study (4/7; 57.1%) had early-stage (IRIS stage 2) CKD and therefore may not have developed secondary hypercalcitoninemia.

Osteoblast-mediated release of ALP is negatively regulated by calcium (43), however, no relationship between blood iCa and ALP was detected. A significant, strong inverse relationship between ALP activity and serum calcitonin was observed when normocalcemic and hypercalcemic cats with calcitonin concentrations above the limit of detection (>0.9 ng/L) were pooled together. Calcitonin is postulated to inhibit the stimulation of osteoblasts by osteoclasts, thus decreasing bone formation and mineralization (3, 44). This study’s findings support the presence of an inhibitory effect of calcitonin on bone turnover in cats in agreement with studies in other species (9). A similar inverse relationship between ALP activity and plasma calcitonin was reported in a previous study in cats (26), although these changes were observed over time and exclusively involved cats with concurrent naturally occurring hypercalcemia and CKD.

Most of the study’s limitations relate to the measurement of calcitonin using a human rather than a feline immunoradiometric assay. Although there is a canine-specific calcitonin assay (45), no feline-specific assay has been synthesized to date. Nevertheless, there is a 70.54% homology between human and feline calcitonin (46), and this assay has been validated and used in feline studies (25, 26). In addition, the majority of both normocalcemic and hypercalcemic cats had a calcitonin concentration below the lower limit of detection of the assay, similar to the previously published feline studies (25, 26). A further study limitation was related to the measurement of calcitonin in serum rather than heparinized plasma. This could not have been avoided since only residual serum was available from all recruited cats. Serum calcitonin measurement is the standard in human medicine, and the intended use for the assay used in the study is the measurement of calcitonin in serum (47, 48). Although using serum was not inappropriate, we cannot be certain that concentrations measured in serum are similar to those measured in heparin. Finally, although samples were stored for an average storage duration of 416 days at –80°C before analysis, this should not have affected calcitonin concentration since no significant correlation was reported between storage time and calcitonin concentration in similar conditions for a median of 1,055 days (26). Another study limitation was the fact that clinical diagnosis in all cats was based on the clinician’s interpretation of findings at the time of case presentation. Unfortunately, the limited volumes of available residual serum samples precluded us from performing additional diagnostics. However, all diagnoses were made by ECVIM diplomates or diplomates in training, regardless of whether the cat was normocalcemic or hypercalcemic. The in-depth review of each case was also conducted by an ECVIM diplomate, and any cases with incomplete medical records were excluded from the study. It should also be noted that control cats were not clinically healthy. This is a common limitation in clinical research, as it would be unethical to obtain samples from normal, healthy cats without clinical justification. Although we cannot exclude it concretely, there is little evidence to support that these diseases influence calcitonin in cats or other species; indeed, the only relevant literature is limited to human thyroid disease (49, 50). A final study limitation relates to the use of ALP as a marker of bone turnover. Total ALP activity in healthy cats mainly consists of liver-specific ALP followed by bone-specific ALP (BALP) (51). Furthermore, ALP has not been validated for the assessment of bone turnover in cats and the hepatic isoenzyme has a short half-life of only 6 hours (52, 53). Nevertheless, previous studies have used ALP as a marker of bone turnover in humans (27) and cats (28). Measurement of more specific markers of bone cell activity that have been validated in cats such as BALP, a marker of bone formation, as well as deoxypyridinoline and carboxy-terminal telopeptide of type I collagen, both markers of bone resorption (54), were considered, but the limited availability of residual serum samples precluded this.

## Data availability statement

The raw data supporting the conclusions of this article will be made available by the authors, without undue reservation.

## Ethics statement

The animal studies were approved by University of Bristol’s Animal Welfare and Ethical Review Body. The studies were conducted in accordance with the local legislation and institutional requirements. Written informed consent was obtained from the owners for the participation of their animals in this study.

## Author contributions

EM: Data curation, Formal analysis, Funding acquisition, Investigation, Visualization, Writing – original draft, Writing – review & editing. CP: Conceptualization, Formal analysis, Investigation, Resources, Validation, Writing – review & editing. AH: Conceptualization, Writing – review & editing. NF: Conceptualization, Formal analysis, Funding acquisition, Methodology, Supervision, Writing – review & editing.
